# Experiences of People Who Discontinue Long‐Acting Injectable Buprenorphine Treatment Recently Released From Prison in New South Wales, Australia

**DOI:** 10.1111/dar.70096

**Published:** 2026-02-18

**Authors:** Lucy J. D. Peck, Bethany White, Maja Moensted, Michael F. Doyle, Elizabeth McEntyre, Antoni Pazeski, Samuel Lawson, Jillian Roberts, Paul S. Haber, Nicholas Lintzeris, Adrian J. Dunlop, Lisa Maher

**Affiliations:** ^1^ Edith Collins Translational Research Centre, Drug Health Services Sydney Local Health District Sydney Australia; ^2^ Specialty of Addiction Medicine, Faculty of Medicine and Health The University of Sydney Sydney Australia; ^3^ Drug & Alcohol Clinical Research & Improvement Network Sydney Australia; ^4^ Mura Leadership Program Fellow, Speciality of Addiction Medicine The University of Sydney Sydney Australia; ^5^ Worimi Guringai and Wonnarua Researcher Australia; ^6^ Drug & Alcohol Clinical Services Hunter New England Local Health District Newcastle Australia; ^7^ Drug and Alcohol Services South Eastern Sydney Local Health District Sydney Australia; ^8^ School of Medicine & Public Health, College of Health, Medicine and Wellbeing University of Newcastle Newcastle Australia; ^9^ Viral Hepatitis Epidemiology and Prevention Program Kirby Institute, UNSW Sydney Sydney Australia; ^10^ Burnet Institute Melbourne Australia

**Keywords:** custody, long‐acting injectable buprenorphine, post‐release, qualitative, retention

## Abstract

**Introduction:**

Among other benefits, opioid agonist treatment (OAT; methadone and buprenorphine) is protective against overdose for people with opioid use disorder following release from custody. Long‐acting injectable buprenorphine (LAIB) is safe with an apparent low risk of diversion relative to other OAT formulations in correctional settings and may become a preferred OAT formulation in this setting. Factors that influence retention in LAIB post‐release are unknown.

**Method:**

People receiving LAIB in nine New South Wales prisons were consented to participate in a survey designed to explore treatment experiences post‐release. Participants who reported ceasing LAIB within 12 weeks of release were invited to complete an in‐depth interview using a semi‐structured guide via phone or secure videoconference in the community, or in custody if reincarcerated. Interviews were transcribed verbatim, and data analysed using iterative categorisation and thematic analysis.

**Results:**

A total of 31 participants who ceased LAIB within 12 weeks following release from custody were interviewed. Mean age was 34 years (SD 9), 24 were male and 16 identified as Aboriginal People. Key themes regarding treatment cessation were misinformation and lack of treatment education, limited transition supports for housing, mental health and illicit substance use, and changing treatment benefits between the custody and community contexts including illicit substance preferences and side effects.

**Discussion and Conclusions:**

Numerous personal experiences and psychosocial factors impacted participant motivations and capacity to continue LAIB post‐release. Understanding and addressing these factors is necessary to support treatment retention and increase community‐based program acceptability and continuation in this population.

## Key Point

- Misinformation about long‐acting opioid treatment impacts retention.
- Factors impacting retention in long‐acting opioid treatment were comparable to other pharmacotherapies.
- Treatment motivation may change post‐release, impacted by illicit substance access.
- Patient education and psychosocial supports are important for long‐acting opioid treatment continuation.

## 1 Introduction

Opioid dependence is estimated to affect over 16 million people globally [1], and is associated with multiple adverse health and societal outcomes, including overdose mortality, blood‐borne virus infection, homelessness and criminal justice system involvement [2, 3]. In the United States (US), individuals experiencing incarceration have significantly higher rates of opioid use disorder (OUD) compared to the general population (8.5% vs. 0.8%) [4] and in Australia, approximately 37% of people with OUD will experience a period of incarceration during their lifetime [5].

The period following release from custody is associated with significantly elevated risk of drug‐related mortality and suicide, demonstrated in both the global [6] and Australian [7] context. Retention in opioid agonist treatment (OAT) during the post‐release period is associated with reduced mortality [7, 8], reduced opioid use [9] and reduced contact with emergency services [10, 11]. However, discontinuation of OAT post‐release is common [12, 13] likely due to transition between OAT providers and heightened psychosocial challenges for people with OUD [14]. Barriers to treatment continuation include service accessibility, homelessness, competing administrative priorities, fragmented transitional support, stigma, lack of family support, relationship and mental health stressors, and return to substance use [15, 16].

Prior to 2020, methadone and sublingual buprenorphine‐naloxone (SL‐BNX) were the primary OAT formulations available in Australian custodial settings [17], however, both have significant potential for diversion [18] and their requirement for daily administration imposes significant resource demands in these environments [19]. A 2019 open‐label trial of the safety and tolerability of long‐acting injectable buprenorphine (LAIB) in New South Wales (NSW) prisons found a reduction in self‐reported opioid use and injecting drug use, with 92% retained at week 16 [20], and strong support from participants [21] and custodial staff [22]. The use of LAIB for OUD in custody in NSW has since increased rapidly, with 96% of patients receiving OAT in custody prescribed LAIB in 2022 [23].

A recent systematic review found that while studies were heterogeneous, LAIB for incarcerated populations is safe, feasible, acceptable and well‐tolerated, and may improve retention post‐release [24, 25]. Two US qualitative studies examined perceptions of LAIB among people in prisons, finding concerns including potential injection site pain [26, 27], limited information about LAIB [26, 27] and safety [26]. These studies, however, interviewed participants retained on treatment [27] or a mixed OAT cohort post‐release [26], limiting exploration of reasons for cessation of LAIB post‐release. Qualitative research in people prescribed LAIB in community settings noted factors impacting LAIB retention included side effects [28, 29, 30, 31, 32], opioid withdrawal [28, 29, 30, 32], return to illicit substance use [28, 32], poor experiences with health care services [32], difficulty with appointment attendance [28], mental health issues [28, 31, 32], insufficient treatment information and scepticism regarding LAIB [30, 31] and reduction in psychosocial supports [29]. These factors may also be relevant for post‐release populations.

Three small‐scale US studies evaluated retention in LAIB post‐release. A trial that randomised OAT prior to release (*n* = 52) from a New York City jail observed higher retention in LAIB 8 weeks post‐release compared to SL‐BNX (69.2% vs. 34.6%) [33]. A retrospective cohort study in Rhode Island examining the use of LAIB during custody and post‐release (*n* = 54) found 30% received at least one community‐based LAIB injection [34]. A larger study (*n* = 200) in Maine found that those released from custody on LAIB were three times more likely than those released on SL‐BNX from a comparison jail to continue their treatment in the community within 35 days of release (67% vs. 23%) [35]. However, these studies lack long‐term follow‐up beyond initial prescriptions being filled post‐release, with reasons for cessation of LAIB not explored.

To our knowledge, reasons for LAIB discontinuation pertaining specifically to the high‐risk period following release from custody have yet to be explored. We therefore aimed to describe the experiences of people prescribed LAIB in custody in NSW who ceased treatment post‐release and explore the barriers to LAIB continuation.

## 2 Methods

### 2.1 Sampling and Recruitment

Adults aged ≥ 18 years old receiving OAT (either methadone or LAIB) in nine NSW correctional centres for a minimum of 6 weeks were consented during routine pre‐release planning procedures by Justice Health and Forensic Mental Health Network (JHFMHN) staff and enrolled in a quantitative survey‐based study which aimed to examine OAT continuation, psychosocial support and substance use 12 weeks post‐release (median 94 days [interquartile range 40; range 79–206]). Of the 243 participants surveyed, 201 had received LAIB in custody. Of the 53 who reported discontinuing OAT, 36 completed an in‐depth interview, of whom four had switched to SL‐BNX and one had received three monthly doses (i.e., had received 12 weeks of treatment in community, the pre‐defined period of interest) and were excluded from the current analysis. Of the 17 not interviewed, six were unable to be contacted again and 11 were not interviewed upon reaching data saturation. The number of Aboriginal people and women was monitored during recruitment to ensure adequate sampling of these subgroups.

### 2.2 Data Collection and Procedures

Interviews were conducted by three authors (LP, SL, AP) and an additional research officer using a semi‐structured schedule to explore issues related to barriers and facilitators of LAIB retention post‐release, prompting discussion around life experiences prior to incarceration, treatment experience in custody, challenges in the post‐release period, and psychosocial factors. The interviewers (one female, three male) were research officers employed by NSW Health with two or more years' experience in the drug and alcohol field. The interviewers had no clinical relationships with participants and personal interaction with participants was limited to a maximum of two phone calls: administering a survey and conducting the in‐depth interview. Interviews were conducted in the community (*n* = 19) or in custody (*n* = 12) if reincarceration had occurred, via telephone (*n* = 23), videoconferencing (*n* = 7) or face‐to‐face (*n* = 1). Interviews lasted 15–50 min, were audio recorded, and transcribed verbatim using professional audio transcription services. Female participants were interviewed by the female researcher (LP) where possible to reduce potential gender‐based power dynamics and encourage participant comfort and openness. As a research team, we engaged in ongoing reflexive practice throughout the study. We acknowledge that our professional roles, gender, and institutional affiliations may have influenced how participants responded to questions, particularly in the context of incarceration and substance use. We discussed these dynamics during team meetings and considered how our assumptions and positionalities shaped data collection and interpretation. The inclusion of Aboriginal and non‐Aboriginal researchers as part of the research team, as well as experienced qualitative, epidemiological and clinical researchers, contributed to a more nuanced and critical analysis of the data.

### 2.3 Data Analysis

Transcripts were analysed, and themes identified using iterative categorisation, a well‐established and widely cited technique for analysing qualitative data developed within the addictions field [36, 37]. Transcription files underwent an initial process of open, deductive coding, with data extracts assigned to a set of codes by lead author (LP) based on the interview guide and the relevant literature [36]. Deductive codes were examined, analytic processes were replicated, and codes were supplemented with inductive codes derived from emergent topics related to the study aims. The second stage involved the interpretive analysis of the data, where data patterns were used to identify recurring codes among the participant population, allowing these ideas to be categorised into higher order concepts and reveal emerging themes. Additional literature was sought and examined to ensure direct links between the analysis and established knowledge [36]. This analytic method allowed for the large amount of textual data to be thoroughly reviewed in a process of descriptive analysis, followed by a process of interpretative analysis to ensure that the themes presented and analysed were rigorously explored and appropriately reflected the experiences of participants. The core research team (LP, BW, AP, SL, AD and LM) met regularly throughout data collection and analysis to discuss the data and emerging themes. Preliminary findings were also disseminated to the wider group to allow co‐authors to interrogate the themes and provide additional feedback to support interpretation using clinical and policy‐based insights.

### 2.4 Ethics Statement

The Release Study, including this qualitative component, was approved by the Hunter New England Local Health District Human Research Ethics Committee (HREC) (X23‐0190 and HREC 2022/ETH01183), the JHFMHN HREC (G968/21; 2021/ETH01010), and the NSW Aboriginal Health and Medical Research Council HREC (1856/21). The project was guided by an Aboriginal Reference Group and this paper has Aboriginal co‐authors. Participation was voluntary and written, informed consent was obtained from all participants.

## 3 Results

### 3.1 Participant Characteristics

Thirty‐one participants were interviewed a median of 123 days (interquartile range 87; range 83–238) post‐release. Participant characteristics including age and gender were included in the results section to both frame extracts and to provide context relevant to social determinants of health that have been shown to impact OUD treatment experience. We have included the Aboriginal and/or Torres Strait Islander status of participants in the results as this is an important determinant of health inequity in Australia, with mental health and substance use disorders key contributors to the burden of disease in this population (24%) [38]. Most participants (*n* = 26; 84%) commenced LAIB while in custody during their recent sentence, receiving a median of 204 days treatment (interquartile range 197; range 35–793) prior to release; 15 for whom it was their first episode of treatment. Over half (61%, *n* = 19) ceased treatment immediately post‐release, with the remaining 39% (*n* = 12) receiving one or two doses of monthly LAIB in community prior to cessation (Table 1). There were no discernible differences in participant demographics or post‐release experiences between participants who received no treatment in community compared to those who received some treatment.

**TABLE 1:** Participant demographics, opioid agonist treatment experience and recent prison history.

Characteristics	*n* = 31
Age, years, mean (SD)	33.7 (9.4)
Gender	
Female (%)	7 (23)
Male (%)	24 (77)
Identify as Aboriginal and/or Torres Strait Islander	
Yes (%)	16 (52)
No (%)	15 (48)
Interview setting	
Community (%)	19 (61)
Custody (%)	12 (39)
OAT experience prior to recent custody sentence	
Continued OAT from community (%)	5 (16)
Commenced LAIB in custody, prior OAT experience (%)	11 (35)
Commenced LAIB in custody, no prior occasion of OAT treatment (%)	15 (48)
Duration of LAIB treatment initiated in custody (*n* = 26)^a^	
Days of treatment received, median, (IQR: range)	204 (197: 35–793)
LAIB activity post‐release	
No treatment received (%)	19 (61)
One dose received prior to cessation (%)	8 (26)
Two doses received prior to cessation (%)	4 (13)
Incarceration history (*n* = 30)^b^	
Length of recent sentence, months, mean (SD: range)	19.7 (14.2: 6–60)

Abbreviations: IQR, interquartile range; LAIB, long‐acting injectable buprenorphine; OAT, opioid agonist treatment.

^a^ Treatment duration extracted from the Justice Health and Forensic Mental Health Network medical record.

^b^ Survey data missing for one participant.

### 3.2 Treatment Misinformation and Lack of Education

Misinformation was perpetuated by and across numerous community networks in participants' lives. This theme was apparent among participants with and without previous OAT experience and encapsulated several recurring points of misinformation related to the health impacts and efficacy of LAIB. Participants with no prior OAT experience were more likely to cite misinformation and lack of education as a reason for LAIB cessation.

Participants identified concerns regarding the efficacy of LAIB. George, a 47‐year‐old Aboriginal man with previous OAT experience, stated his peers were concerned that because LAIB does not include a “blocker” [opioid antagonist] such as naloxone, the treatment would not stop him from using illicit opioids, and therefore discouraged him from continuing:

I felt totally secure but then I was telling my mate … and he said the Buvidal injection doesn't stop you using, it's not like the Suboxone … it doesn't actually block the fucking opioid—George.

Ben, a 38‐year‐old Aboriginal man with previous OAT experience, acknowledged that he did not understand ‘the science behind it [LAIB]’. He believed that continual use of LAIB would lead to a tolerance, similar to his experience with long‐term use of illicit substances, and therefore felt the medication was pointless:

Just doesn't stay in the system, I don't think for that long, it wears off pretty quick … It doesn't matter who you are, you keep taking a cap [of drugs] a day, a cap's not going to do nothing to you … And I'm guessing that's the same with the injection … so what's the point in taking it?—Ben.

Two participants identified pregnancy as a reason for treatment cessation and described concerns expressed by family and community creating fear and uncertainty about the health impacts. This is illustrated in the case of Emma, a 21‐year‐old Aboriginal woman with no previous OAT experience, who was released from custody on LAIB approximately 30 weeks pregnant:

I was eight months pregnant. [I] was going to do my Buvidal injections and then everyone scared me saying ‘you need to get off it or your daughter is going to be affected’, so it scared me, and I jumped off the injection after my first dose in community—Emma.

Similarly, Mary, a 22‐year‐old non‐Aboriginal woman with no previous OAT experience, relayed that upon discovering her pregnancy 2 weeks after release, she became fearful of continuing treatment due to the advice from family members. Mary did not receive any advice from medical professionals regarding LAIB during pregnancy. This absence of information resulted in the opinions of trusted family members being weighted heavily:

My dad … He told me to stay off it when I was pregnant. He didn't say why I should do it. [Was there anyone else providing you with information?] No—Mary.

Misinformation regarding the health impacts of LAIB from peers and family members and inadequate education regarding LAIB highlight a gap in understanding LAIB among this cohort and subsequent scepticism regarding treatment.

### 3.3 Changing Treatment Benefits Between Custodial and Community Contexts

Several participants indicated that they had ceased treatment because LAIB did not provide them with the same benefits in the community as it did in custody. Tracey, a 27‐year‐old Aboriginal woman with previous OAT experience, returned to substance use immediately post‐release:

I found it a lot more beneficial when I was inside than when I was outside … when you're on the outside it's a lot easier to access [drugs] and you kind of go more in that direction—Tracey.

This was related to a widely ascribed motivation for seeking LAIB treatment in prison. Several participants mentioned non‐prescribed SL‐BNX was the mostly commonly available illicit substance in prison, spending up to $1000 Australian dollars (AUD) for a strip of SL‐BNX film in prison and sharing injecting equipment. In NSW prisons, access to safe injecting equipment through Needle Syringe Programs is prohibited as in other Australian jurisdictions [39]. Lowering costs and reducing the risk of bloodborne viruses in custody was a key motivator for Michael, a 21‐year‐old Aboriginal man with no previous OAT experience, who sought LAIB in custody primarily to avoid these environment specific issues:

I only got on [LAIB] because I was sick of using needles in jail, I was sick of shooting up, wasting money in jail. I just wanted to get off it because when there was nothing left in the pot, I was sick of hanging out, so I had to get on the program for something to help me—Michael.

Once released, participants had access to safe injecting equipment and a wider range of illicit substances. A history of polysubstance use was also common among participants. Shaun, a 28‐year‐old non‐Aboriginal man, and Simon, a 35‐year‐old non‐Aboriginal man, noted a preference for methamphetamines or cannabis over opioids:

When you get out you jump off the bupe and just get on the crack [methamphetamine] … They [OTP clinic staff] tried to tell me I'm an idiot [for coming off LAIB] and that I'm gonna come down and that I'll lose myself and I wouldn't be able to work. Well, I guess they don't understand the power of meth—Shaun

[Why have you gone for the medicinal marijuana over Buvidal?] Just health reasons … When I smoke weed, I reckon it's more healthier, it's more natural and I've smoked weed my whole life—Simon.

For participants such as Shaun and Simon, illicit opioids were the only available substance in prison, and accessing LAIB enabled the temporary management of substance use while in custody to avoid negative financial and health impacts. These participants both had no previous OAT experience and noted that LAIB and using other opioids were no longer desirable post‐release once they were able to access preferred substances.

Participants also discussed the side effects of LAIB as a deterrent to continuing treatment in the community. Some participants attributed decreased libido and erectile dysfunction to LAIB, a side effect possibly related to opioid‐induced androgen deficiency observed with chronic opioid use [40] and previously observed in patients receiving SL‐BNX [41]. This side effect was less concerning for participants while in custody; however, it caused significant treatment dissatisfaction in community. Thomas, a 31‐year‐old Aboriginal man with no previous OAT experience, was not concerned about the impact on his libido in custody; however, things changed post‐release:

It's more easy and kicked back in jail because you're pretty much confined to a cell. You're sitting around stoned on the fucking Buv [LAIB] … but being on it you don't have a high sex drive, you don't have all that shit so you're not really fucked in jail but when you're out it's not a good look—Thomas.

Participants like Lucas, a 41‐year‐old non‐Aboriginal man, was also disillusioned with LAIB due to the impact on his libido post‐release. Lucas, who had previous OAT experience and had noted similar impacts on sexual function with methadone, felt that LAIB comparatively did not provide enough of a benefit in community to outweigh the impacts on libido:

It [LAIB] fucks with your sex drive. Yeah, okay methadone fucks with your sex drive but at least it's an actual opiate … This stuff's an artificial thing—Lucas.

Differences between the custodial and community contexts significantly impacted participant satisfaction with LAIB. The benefits afforded by LAIB in relation to managing opioid dependence were sometimes no longer relevant or not sufficient post‐release to support LAIB retention.

### 3.4 Limited Transition Supports in the Post‐Release Period

Consistent with treatment retention challenges reported by persons released on other forms of OAT [14, 16], as well as previous literature examining barriers to LAIB retention among non‐custodial populations [29, 30], a strong pattern emerged among participants regarding a lack of stability and support as a major barrier to continuing LAIB following release from custody. Participants generally felt that they were poorly linked with services or were not sufficiently supported.

The transition from being institutionalised to ‘adjusting to independence’ led some participants to feel shocked and overwhelmed upon release from custody. Colin, a 27‐year‐old Aboriginal man with no previous OAT experience, felt that LAIB dosing was an additional burden that was difficult to manage due to competing priorities:

When you get out, you're back into society and you've got to sort out money, you've got to sort out this and sort out that. There's just so much. I know I was doing it beforehand but after nearly two years in jail of not needing to do anything, coming back out it's like, ‘What do I do?’—Colin.

Participants like Colin struggled with the sudden isolation, independence and self‐accountability that came with release and felt they needed more support during this critical transition period. Harry, a 49‐year‐old non‐Aboriginal man with previous OAT experience, felt that the accountability provided by regular check‐ins from support persons may have enabled him to continue LAIB in community:

[You didn't have a parole officer?] No, no one was checking up on me … maybe if I had a bit of supervision, I might be a bit different now—Harry.

It should be noted that the desire for increased parole‐related supervision was uncommon among participants, with several reporting that their parole officers were “useless” or deliberately obstructive. However, some participants noted the potential benefit being on parole, which while focused on conditions related to a reduce custodial sentence rather than necessarily facilitating health service referrals, could provide support with accessing housing and employment. Caleb, a 39‐year‐old non‐Aboriginal man with previous OAT experience, shared his experience with parole as negative and obstructive, leading to further mistrust of service providers commonly experienced by this population [42] and ultimately disengagement from treatment:

My parole officer that was in charge of me didn't give two fucks of what I said to her … and she done the total opposite of what I've sat down and made a case plan with her about—Caleb.

Participant experiences with parole services did not necessarily directly lead to treatment cessation, however highlighted a wider issue related to the general lack of support received, as well as feelings of disrespect and dismissal by such services.

Our findings suggest that psychosocial challenges in the post‐release period including mental health decline and illicit substance use were not adequately addressed, resulting in disengagement from treatment, as previously reported with other forms of OAT post‐release [15, 16] and among general populations receiving LAIB [28, 31, 32]. This was noted by Mia, a 29‐year‐old non‐Aboriginal woman with previous OAT experience, whose return to polysubstance use impacted her treatment goals:

I ended up relapsing on to ‘ice’ [crystal methamphetamine] and heroin and I didn't get to follow up with my treatment, which then spiralled down into it and then back onto the same road that I was before—Mia.

Downward trends in mental health post‐release resulted in participants such as Anthony, a 49‐year‐old non‐Aboriginal man with previous OAT experience, feeling overwhelmed by the task of attending the clinic for LAIB:

[Can you tell me why you stopped taking LAIB?] I think it was mainly just my mental health. I just felt overwhelmed. Yeah, it was one extra burden that I couldn't afford to have—Anthony.

Anthony noted that he had previously struggled to maintain his treatment on methadone due to similar mental health challenges. Despite the significantly reduced time commitment afforded by monthly LAIB dosing, this issue persisted as a barrier to treatment retention when his mental health was not addressed.

Several participants felt that access to housing was the ‘biggest challenge’ and was a ‘major barrier’ to retention in community‐based LAIB treatment, with housing stress decreasing capacity to maintain treatment goals and avoid illicit substance use. Caleb, introduced previously, discussed how temporary accommodation was a major barrier due to exposure to others who had a negative impact on mindset or treatment goals:

I was doing so well on this fucking program … my headspace was pretty strong, I was in a good place … so I asked them to be put in a place and not go to a fucking halfway house ‘cause I didn't want to surround myself with fuckwits again—Caleb.

For participants such as Anthony, finding suitable accommodation took up a significant amount of time and energy and greater support accessing housing may have increased capacity to focus on health:

[What would have needed to be different for you to stay on the LAIB program?] More secure housing … I could have focused a bit more on medication but it just didn't work out that way—Anthony.

Several participants identified that more intensive support and supervision from specialised services such as the Connections Programme, a pre and four‐week post‐release support service managed by JHFMHN, may have helped them remain in LAIB treatment. The Connections Programme provides caseworker support navigating social and health service post‐release to a limited number of people released from NSW prisons. Participants such as Caleb, who had access to the Programme, felt the service was a ‘great support’ but was not long enough:

I had Connections and that was it … but the lady herself that was helping me, she was fucking great! But it only lasted a month—Caleb.

Despite the reduction in required clinic attendance afforded by LAIB, it was evident that participants still faced difficulties attending appointments and lacked sufficient support between and outside dosing attendance. Participants felt that additional factors improving quality of life including mental health support and housing would facilitate ongoing capacity to remain in treatment.

## 4 Discussion

To our knowledge, this research is the first to provide insights into the barriers to LAIB continuation following release from custody. As the number of opioid dependent persons prescribed LAIB in Australian custodial settings increases [23] and the feasibility and effectiveness of LAIB in correctional populations continues to be demonstrated globally [24, 25], it is important to better understand the barriers to treatment retention post‐release to improve patient outcomes.

Misinformation surrounding the efficacy and safety of LAIB among participants was a clear theme that led to treatment scepticism and ultimately cessation. Concerns regarding the lack of naloxone in LAIB, uncertainty regarding the long‐acting nature of LAIB, tolerance development and a general lack of understanding regarding LAIB pharmacology ultimately led some participants to discontinue treatment. Our findings are consistent with previous research in other populations discontinuing LAIB that reported insufficient information regarding the medication [31] and hesitation due to being a newer and ‘insufficiently researched’ medication [30]. Misinformation regarding LAIB was also perpetuated by family and peers, exacerbated by limited access to medical advice from trusted professionals. Participants who experienced pregnancy while on LAIB cited fear regarding the safety of the foetus as a reason for LAIB cessation. While subsequent research has supported the safety of LAIB during pregnancy for both maternal and newborn health [43, 44], this is perhaps unsurprising given the relative lack of evidence available at the time this work was conducted. Limited treatment information for LAIB has been noted among incarcerated populations pre‐ and post‐release [26, 27], with our findings supporting this issue as a reason for treatment cessation post‐release. Overall, these findings suggest people new to treatment with limited experience of LAIB or other OAT may benefit from additional prerelease education and targeted post‐release support. Of note, the impact of limited information was evident among pregnant participants. Previous research has shown that pregnancy magnifies existing barriers to accessing OAT post‐release [45], with additional challenges including stigma from health care professionals and family, fear of involvement with child protective services, and negative influence from abusive partners [46]. There is currently limited evidence on the experience of pregnant people receiving LAIB, and while this issue warrants further exploration, no other potential gender differences in experiences emerged from our data.

We also observed a change in treatment motivation between custody and community settings. Illicit substance cost and availability in prisons has been cited by previous Australian research examining motivation for seeking OAT in custody [47, 48]. Our findings suggest this may also be a reason for LAIB treatment cessation, with return to substance use also previously reported as a reason for discontinuing other forms of OAT post‐release [16]. Participants with non‐opioid substance preferences noted that in custody, LAIB enabled them to avoid negative financial and health impacts but, once released from custody, expressed no interest in continuing LAIB due to a perceived lack of need. The impact of medication side effects, namely decreased libido and erectile dysfunction, also varied between settings. Reduced satisfaction with LAIB post‐release illustrates changing priorities between the custody and community contexts, with the negative impacts of unwanted side effects in community outweighing LAIB benefits. The impact of differing treatment motivation between custodial and community contexts on LAIB continuation post‐release warrants further investigation, including alternative supports to OAT in community.

This study confirms that some of the structural and psychosocial barriers reported with daily methadone and SL‐BNX continuation post‐release are similarly experienced by patients released on monthly LAIB. Despite reduced time required for clinic attendance, housing, mental health support, and substance use support appeared to have similar impacts on patient capacity to engage in treatment post‐release, as reported with those released on methadone or SL‐BNX [15, 16, 49, 50]. Further, mental health issues, lack of psychosocial supports, homelessness and a return to illicit substance use have been shown to contribute to LAIB discontinuation generally [28, 30, 31, 32, 50], suggesting consistencies between general and post‐release populations. A desire for more intensive support for longer periods post‐release was common. Previous research in non‐custodial populations found that participants on LAIB often felt unsupported by the OTP services due to reduced contact with clinicians created by monthly dosing schedules [48, 51], with findings highlighting the importance of robust wrap‐around supports for LAIB patients [30]. Less engagement and support from OTP staff undermined patient capacity to achieve goals separate to LAIB adherence (e.g., mental health improvements, gaining employment) [52]. Similarly, OTP staff perspectives have identified the issue of reduced patient contact with monthly LAIB, noting it made it difficult to form relationships with patients and provide the support needed [29]. Both previous research and the current findings may indicate that the reduced contact with OTP staff resulting from monthly dosing may not be suitable for all patients, particularly those facing psychosocial issues likely benefiting from additional outreach/support. Previous research has highlighted the benefits of intensive and personalised case management post‐release to support OAT retention and improve other health outcomes [53, 54, 55, 56]. With regard to mental health challenges, consideration should also be given to OAT treatment type post‐release, as some patients with comorbid psychopathologies may be better supported with high dose full agonists such as methadone [57, 58]. However, more recently, positive experiences of LAIB in community settings among cohorts with complex psychiatric comorbidities have also been noted [59].

Several limitations should be considered when interpreting these findings. Our study explored the experiences of people recently released from custody with negative or dissatisfactory experiences of LAIB and specifically the experiences of those who did not continue LAIB post‐release. Exploring the experience of participants who continued LAIB and the enablers to treatment retention could help further shape the interpretations of these results. Findings are not generalisable to other groups, and the complexity and nuance of post‐release LAIB experiences of this population should be considered when interpreting results. Further research in this area needs to occur to determine whether our findings are observed in other settings. Our analysis also relied on self‐report data from participants, which may be impacted by social desirability bias and recall bias.

## 5 Conclusions

We found that the potential of LAIB to enhance convenience may not be realised for some people and that LAIB is not a panacea for addressing the social, structural and ecological issues surrounding treatment engagement and retention following release from custody. With the recent significant upscaling of LAIB prescription for opioid dependent people in custody in Australia, adapting models of care to address factors impacting ongoing treatment engagement following release has the potential to improve continuity of care. Identifying higher‐risk groups prior to release, particularly those with limited OAT experience or those likely to face increased psychosocial and structural challenges in the community, may increase LAIB retention. This may be achieved through targeted provision of treatment education and individualised post‐release support planning within a more person‐centred model of LAIB care that accounts for patients' treatment experiences, personal preferences and environmental factors upon release. Exploring these barriers to LAIB retention post‐release is important for understanding the needs of this cohort and building a more comprehensive model of care that prioritises treatment satisfaction and patient wellbeing.

## Author Contributions

Lucy J.D. Peck: formal analysis, data curation, investigation, writing – original draft. Bethany White: conceptualisation, methodology, investigation, data curation, funding acquisition, writing – review and editing, supervision, project administration. Maja Moensted: methodology, data curation, writing – review and editing. Michael F. Doyle: conceptualisation, methodology, funding acquisition, writing – review and editing. Elizabeth McEntyre: conceptualisation, methodology, writing – review and editing. Antoni Pazeski: investigation, writing – review and editing. Samuel Lawson: investigation, writing – review and editing. Jillian Roberts: conceptualisation, methodology, funding acquisition, investigation, resources, writing – review and editing. Paul S. Haber: conceptualisation, methodology, funding acquisition, investigation, resources, writing – review and editing. Nicholas Lintzeris: conceptualisation, methodology, funding acquisition, investigation, resources, writing – review and editing. Adrian J. Dunlop: conceptualisation, methodology, funding acquisition, resources, supervision, data curation, project administration, investigation, writing – review and editing. Lisa Maher: conceptualisation, methodology, writing – review and editing, supervision.

## Funding

This work was supported by the NSW Health Translational Research Grants Scheme.

## Conflicts of Interest

This work was supported by the NSW Health Translational Research Grants Scheme, Medical Research Future Fund (P.S.H.) research fellowship and National Health and Medical Research Council (L.M., M.D.) research fellowships. A.J.D. reports grants from Camurus AB and Indivior to conduct clinical studies with buprenorphine formulations to Hunter New England Local Health District, his employer. P.S.H. reports grants from Camurus AB and Indivior to conduct clinical studies with buprenorphine products to SLHD, his employer, and has served on advisory boards for Lundbeck, Indivior, AbbVie and Gilead. N.L. reports grants from Camurus AB to conduct sponsored and investigator‐led clinical studies with long‐acting injectable buprenorphine to South Eastern Sydney Local Health District, his employer, and has received honoraria from Camurus for leading professional education sessions. All other authors declare no competing interests.

## Data Availability

The data that support the findings of this study are available on request from the corresponding author. The data are not publicly available due to privacy or ethical restrictions.
